# Learning biologically-interpretable latent representations for gene expression data: Pathway Activity Score Learning Algorithm

**DOI:** 10.1007/s10994-022-06158-z

**Published:** 2022-04-29

**Authors:** Ioulia Karagiannaki, Krystallia Gourlia, Vincenzo Lagani, Yannis Pantazis, Ioannis Tsamardinos

**Affiliations:** 1 Institute of Electronic Structure and Laser, Foundation for Research and Technology-Hellas (IESL-FORTH), Heraklion, Greece; 2 Department of Computer Science, University of Crete, Heraklion, Greece; 3 Institute of Chemical Biology, Ilia State University, Tbilisi, 0162 Georgia; 4 JADBio, Gnosis Data Analysis PC, Heraklion, Crete Greece; 5 Institute of Applied and Computational Mathematics, Foundation for Research and Technology - Hellas, Heraklion, Greece

**Keywords:** Pathway activity, Dimensionality reduction, Disease classification, Differential activation analysis

## Abstract

Molecular gene-expression datasets consist of samples with tens of thousands of measured quantities (i.e., high dimensional data). However, lower-dimensional representations that retain the useful biological information do exist. We present a novel algorithm for such dimensionality reduction called Pathway Activity Score Learning (PASL). The major novelty of PASL is that the constructed features directly correspond to known molecular pathways (genesets in general) and can be interpreted as *pathway activity scores*. Hence, unlike PCA and similar methods, PASL’s latent space has a fairly straightforward biological interpretation. PASL is shown to outperform in predictive performance the state-of-the-art method (PLIER) on two collections of breast cancer and leukemia gene expression datasets. PASL is also trained on a large corpus of 50000 gene expression samples to construct a universal dictionary of features across different tissues and pathologies. The dictionary validated on 35643 held-out samples for reconstruction error. It is then applied on 165 held-out datasets spanning a diverse range of diseases. The AutoML tool JADBio is employed to show that the predictive information in the PASL-created feature space is retained after the transformation. The code is available at https://github.com/mensxmachina/PASL.

## Introduction

Molecular data, such as gene expressions, are often very high dimensional, measuring tens of thousands molecular quantities. For example, the Affymetrix microarray platform GPL570 for humans measures the expression of 54675 probe-sets, corresponding to all known human genes. As such, visually inspecting the data, understanding the multivariate gene correlations, and biologically interpreting the measurements is challenging. To address this problem, several methods have appeared that reduce the dimensionality of the data. Dimensionality reduction (a.k.a. latent representation learning) constructs new dimensions (features, quantities, variables) with the purpose of reducing the number of features, making them amenable to inspection while maintaining all “useful” information. For example, consider the representation of music. The raw data (original measured quantities) correspond to the sound spectrum which is visually incomprehensible to humans. However, music at each time-point can be represented as a sum of prototypical states (notes) and musical scores, which are much more intuitive. Similarly, we can ask the questions: Are there prototypical cell states whose sum can represent any cell state (i.e., gene expression profile)? What are the “notes” of biology? How can we learn such representations automatically?

Numerous dimensionality reduction techniques have been proposed. Some of the most prevalent ones are arguably the PCA (Abdi and Williams, 2010), Kernel PCA (Schölkopf et al., 1998), t-SNE (Maaten and Hinton, 2008), and Neural Network autoencoders (Chicco et al., 2014; Danaee et al., 2017). All of these methods learn a lower dimensional space (latent space) of newly constructed features and represent the data as a linear or non-linear combination of those. The projection aims to retain the data variance and exhibit a low data reconstruction error. However, the data representation in the new feature space is biologically unintepretable. To improve interpretability, other methods introduce sparsity to the latent space in the sense that new features are constructed as a linear combination of only a few of the original molecular quantities. Such methods are the Sparse PCA (Zou et al., 2006) and sparse variants of Non-negative Matrix Factorization (Lee and Seung, 1999) for molecular data (Carmona-Saez et al., 2006; Fertig et al., 2010). The new constructed features are sometimes called *meta-genes* (Brunet et al., 2004). Any clustering method could also be defined as creating meta-genes and new features. However, *the meta-genes are still hard to interpret biologically as they do not directly correspond to the known biological pathways or other known gene sets*. In contrast, methods like Gene Set Variation Analysis (GSVA, (Hänzelmann et al., 2013)) employ enrichment statistics commonly used in gene set enrichment analysis (Subramanian et al., 2005) for constructing new features. Each new feature corresponds to a different, but known, biological pathway. While GSVA features are undoubtedly more intuitive than the ones derived through general-type dimensionality reduction, GSVA neither explicitly nor implicitly aims to retain the information contained in the original features within the derived features, and the derived features are strictly limited to the pathways specified a priori.

This work, which significantly extends (Karagiannaki et al., 2020) both methodologically and experimentally, proposes a novel method for unsupervised feature construction and dimensionality reduction based on the availability of prior knowledge, called Pathway Activity Score Learning or **PASL**. PASL aims at a trade-off between biological interpretability and retaining all information contained in the original features. PASL accepts as input a collection of predefined sets of genes, hereafter called genesets, such as molecular pathways or gene ontology groups. It has two phases, the *inference phase* and the *discovery phase*. During the inference phase, *PASL constructs new features that are constrained to directly correspond to the available genesets.* Each new feature can be thought of expressing an activity score of its corresponding geneset, summarizing in a single value an aspect of the collective behavior of the geneset’s gene expressions. It is possible however, that several new features correspond to the same geneset (many-to-one relation), expressing different aspects of the collective activity of its genes. The order of insertions of genesets in the dictionary is determined by the amount of variance explained by each atom (i.e., each element of the dictionary), while the estimation of the atom’s coefficients (a.k.a. loadings) is performed through PCA. PASL’s inference phase incorporates heuristics that greatly accelerate the estimation of the dictionary without compromising its representation capacity.

The inference phase ends when it has captured as much information as possible given only the provided genesets. However, a large percentage of the measured quantities is not mapped to any known geneset. In the discovery phase, PASL constructs features that are not constrained to correspond to the given genesets trying to capture the remaining information (variance) in the data. Due to its efficiency, we employ Sparse PCA (Zou et al., 2006) for the estimation of the atoms of the discovery phase.

We evaluate PASL’s ability to represent data in a latent feature space in three aspects: (a) The out-of-sample **percentage of explained variance** (i.e., one minus the relative reconstruction error). Specifically, we measure the percentage of variance explained (maintained) when held-out test data are transformed to the latent space and back to the original gene expression space. However, obtaining a low reconstruction error does not necessarily mean that it is the “*important*” information that is captured and maintained. We define as important the information that helps us predict an outcome of biological or clinical interest, such as the disease status, the response to treatment, or some other quantity of interest. This leads us to use a second metric for evaluating PASL: (b) **the predictive performance** maintained for an outcome of interest in held out datasets. To measure the predictive performance in a gene expression dataset, we apply the automated machine learning (AutoML) tool JADBio (Tsamardinos et al., 2020) (standing for Just Add Data Bio). We compare the predictive performance achieved by JADBio on the original gene expression data against the performance achieved by models trained on the transformed data. To ensure that a high-quality predictive model is built, JADBio searches thousands of machine learning pipelines (called configurations) to identify the optimally predictive ones and estimates the out-of-sample predictive performance of the final model in a conservative fashion. Finally, (c) since a PASL-constructed feature directly corresponds to known geneset, we can consider it as a geneset activity score. The geneset activity scores can be employed to perform **differential activation analysis** (DAA) and identify the genesets that are statistically discriminative between two different classes or conditions (e.g., cases vs controls, or treatment vs controls). Conceptually, this is equivalent to gene differential expression analysis that identifies genes whose expression behaves differently in two classes. We evaluate the statistical power and the *p*-values produced by DAA and compare it with standard gene set enrichment analysis (GSEA).

We perform two main sets of computational experiments on real datasets:

- We evaluate PASL algorithm on two large collections of datasets, one employing datasets related to *Breast Cancer* and the other datasets related to *Leukemia* involving several types of outcomes such as mutation and hormone status, dietary restrictions, responder or non-responder to a specific treatment etc. For each collection 80% of the datasets are used for learning a dictionary (training data) and the rest are employed for testing. We contrast results against PLIER (Mao et al., 2019), arguably the algorithm closer in spirit to PASL. The results here show that *PASL outperforms PLIER in terms of predictive performance on the test sets*. Quite importantly, PASL’s feature space maintains the predictive information. In addition, we show that DAA using the constructed features can complement standard GSEA, in the sense that it can identify genesets that are not identified by GSEA as statistically significant. Moreover, DAA has larger statistical power than GSEA and, in general, it identifies the affected genesets with lower *p*-values than GSEA.
- In the second large-scale experiment, we construct a universal latent space (dictionary) for gene expression data of GPL570 platform. We use 1736 datasets from the Biodataome (Lakiotaki et al., 2018) repository and a subset of 50000 samples, the largest we could fit in memory, to learn the dictionary. 10% of the datasets (165 are kept after excluding the ones without a defined discrete outcome) were held out for testing spanning 25 different disease categories. *The universal dictionary is shown to maintain the predictive information across pathologies and phenotypes*. In several cases, it leads to models easier to interpret biologically and visually.

Overall, the results show that PASL (i) enables compression of the gene expression datasets that lead to 1 order of magnitude speed up in modelling, (ii) maintains the predictive information across pathologies, tissues, outcomes, and phenotypes while often leading to simpler models that are easier to interpret biologically, and (iii) complements standard GSEA in identifying differentially affected genesets across two conditions.

We envision that PASL can help in transitioning gene expression data analysis techniques from a purely gene-centric perspective to a more systemic, pathway-centric approach. While current works focus on selecting *relevant genes* (Kuang et al., 2021) or identifying *gene regulatory networks* (Mignone et al., 2020), applying PASL in combination with these methods may allow to identify *relevant pathways* and *pathway regulatory networks* instead.

## Pathway activity score learning algorithm

### Preliminaries

The PASL algorithm accepts as input two 2D matrices *X* and *G*. Matrix $X\in {\mathbb {R}}^{n\times p}$ contains the molecular measurements, where *n* is the number of samples and *p* the number of features. Typically $n \ll p$. For microarray gene expression data, the rows of *X* correspond to molecular profiles while the columns to probe-sets. Due to multiple technical factors specific to microarray technology, each probe-set can end up measuring the expression value of a single gene, no genes or even multiple genes, and the same gene can be measured by multiple probe-sets as well. For example, the microarray platform used in our experiments, namely the Affymetrix Human Genome U133 Plus 2.0 Array, measures 54675 probes, corresponding to 21299 unique genes. PASL also accepts a gene membership matrix $G \in \{0, 1\}^{m\times p}$ with *m* being the number of predefined groups of genes. Each row of *G*, denoted by ${g}_i$ for the *i*-th row, corresponds to a molecular pathway, gene ontology set, or any other predefined gene collection of interest called geneset hereafter. We set $G_{ij} = 1$ if *at least one* of the genes measured by probe-set *j* belongs to the *i*-th geneset, and 0 otherwise. In this way the *G* matrix can effectively represent cases where multiple genes are measured by the same probe-set. Finally, even though there is not perfect one-to-one correspondence between probe-sets and genes, hereafter we will refer to probe-sets as genes for simplicity, unless otherwise noted.

PASL initially standardizes the data matrix with $X=\frac{(X - \mu )}{\sigma }$ where $\mu \in {\mathbb {R}}^p$ and $\sigma \in {\mathbb {R}}^p$ are the mean and standard deviation vectors of all features and the standardization operation is performed column-wise. Then, it assumes the *standardized* data *X* can be decomposed as:

1 $$\begin{aligned} \boxed {X = L \cdot D + \eta } \end{aligned}$$

where $D\in {\mathbb {R}}^{a \times p}$ is a sparse matrix corresponding to the dictionary with *a* denoting the number of dictionary atoms (i.e., the number of rows), while $\eta$ is an i.i.d. additive noise term. In other words, each molecular profile at row *j* of *X* is a linear combination of rows of *D* with coefficients in the *j*th row of *L* with an isotropic noise added to it. Given training data *X*, PASL outputs the two matrices, *D* and *L*. It also outputs the means $\mu$ and standard deviations $\sigma$ of each feature to allow standardization on future data. *D* is the concatenation of two sub-dictionaries $D_1$ and $D_2$ ($D = [D_1 ; D_2]$) with dimensions $a_1\times p$ and $a_2\times p$, respectively (hence, $a = a_1 + a_2$). $D_1$ *is a dictionary where each atom* *d* *is constrained to correspond to only one geneset of the matrix* *G*, in the sense that the non-zero elements of *d* correspond to the genes in the particular geneset. Thus, $D_1$ is the part of the dictionary that is biological interpretable. $D_2$ is just a sparse dictionary meant to explain the remaining variance of the data and *suggests the existence of yet-to-be-discovered genesets*. $D_1$ is the outcome of the first phase of PASL, called the inference phase, while $D_2$ is the outcome of the second phase, called the discovery phase. $L\in {\mathbb {R}}^{n\times a}$ is the representation of the data in the latent feature space (*PASL scores*). It provides the optimal projection of *X* on the row space of *D* and it is computed by minimizing the Frobenius norm between the normalized *X* and $L\cdot D$ giving raise to the formula:

2 $$\begin{aligned} \boxed {L = X \cdot D^+} \end{aligned}$$

where $D^+$ is the pseudo-inverse of *D*. Before continuing with the detailed description of PASL algorithm, we provide a table (Table 1) with PASL’s variables and their explanation.

**Table 1:** List of PASL algorithm’s variables

Variable	Description	Dimension
*X*	Data matrix	$n\times p$
$\mu$	Mean (row) vector	$1\times p$
$\sigma$	Std (row) vector (per column/feature)	$1\times p$
*G*	Gene membership matrix	$m\times p$
*D*	Dictionary matrix	$a\times p$
*L*	Score matrix	$n\times a$
$\eta$	White noise matrix	$n\times p$
*d*	Atom (i.e., element/row of *D*)	$1\times p$
*u*	(1st) Principal component (eigenvector)	$|\text {Geneset}|\times 1$
$g,g_i,g_k$	Geneset (set)	Varying
$v_{i,j}$	Explained variance (unnormalized)	$1\times 1$
${\tilde{v}}_{i,j}, {\hat{v}}$	Box-Cox norm. explained variance	$1\times 1$
${\mathcal {I}}$	Ordered index set (i.e., a list)	$1\times a_1$
$\tilde{{\mathcal {V}}}$	Ordered set with Box-Cox norm. variance values	$1\times a_1$
*t*	Static vs dynamic trade-off threshold	$1\times 1$
$\lambda$	Box-Cox transformation hyper-parameter	$1\times 1$
*nz*	Number of non-zero elements in Sparse PCA	$1\times 1$

### The PASL algorithm

**Inference Phase**: The inference phase of PASL implements a *greedy* approach that constructs new atoms one at a time. Every next atom *d* to-be-constructed corresponds to a geneset *g* so that *d* has non-zero coefficients only for the genes that belong in *g*. The algorithm needs to address two issues:

1. **What is the geneset** *g* **to be used for the construction of** *d*? The heuristic we propose is *to select the* *g* *that leads to the next atom d that explains the most of the data variance.* To account for the different size of the genesets, the explained variance is normalized for the geneset size according to a Box-Cox normalization.
2. **Given** *g*, **how is the next atom** *d* **constructed?** This part is straightforward. Since we are only allowed to have non-zeros only at the genes of *g*, we perform a standard PCA restricted (i.e., reduced) to the genes of *g*, thus ignoring the rest. The non-zero coefficients of *d* correspond to the respective elements of the first principal component of this reduced PCA. Once *d* is constructed, its contribution to the explained variance is removed from the data and the algorithm re-iterates.

**Fig. 1:**
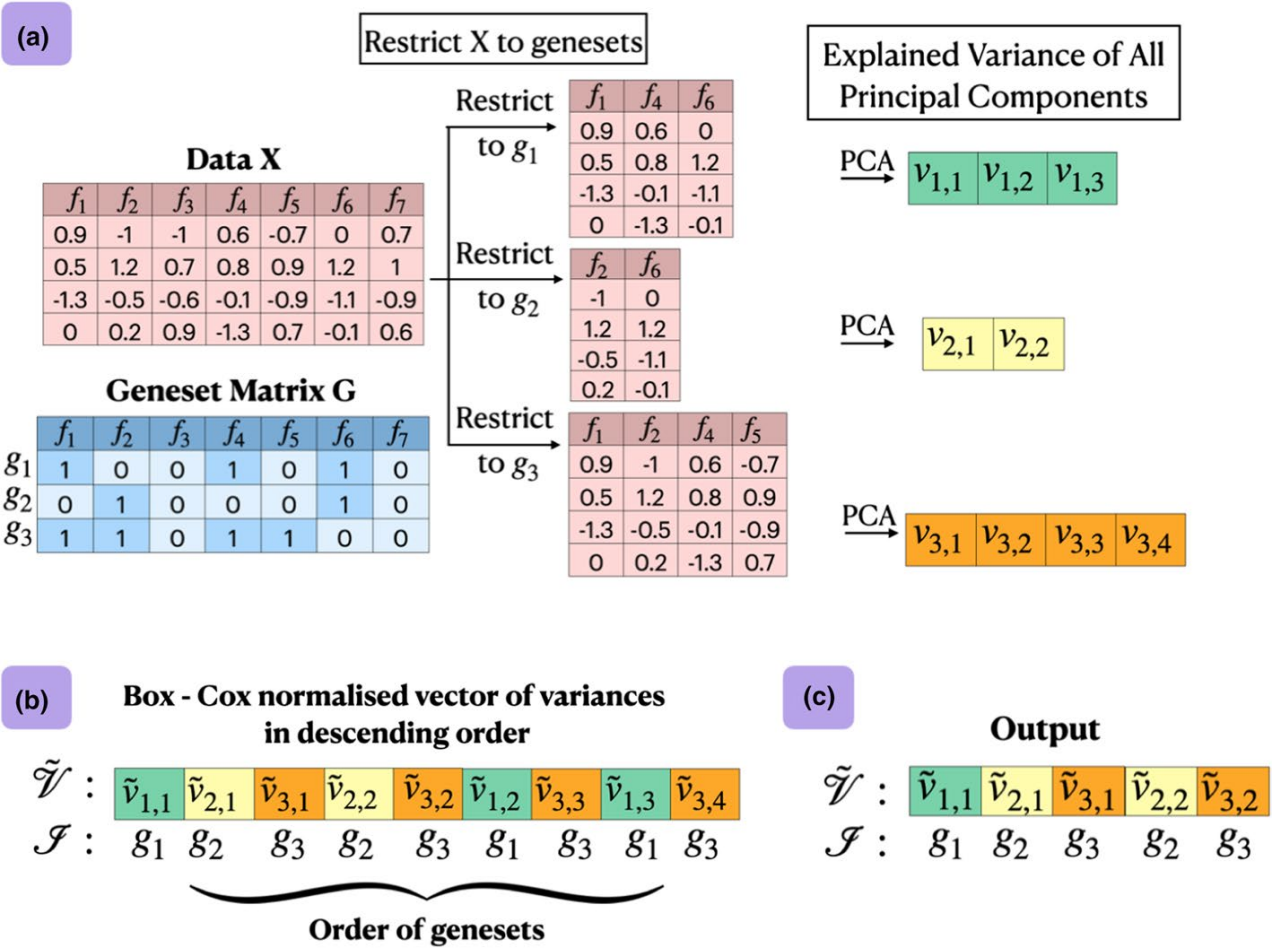
The *OrderOfGeneSets* function (Algorithm 1) returns a heuristically determined order of genesets in the ordered set ${\mathcal {I}}$. **a** The data matrix *X* is shown having $n=4$ samples (rows) and $p=7$ gene expressions denoted with $f_1, \ldots , f_7$. For each geneset $g_i$, a full PCA is performed restricted to the genes in $g_i$. The explained variances for geneset $g_i$ and the *j*-th principal component are denoted with $v_{i,j}$. **b** The variance values are normalized for geneset size using a Box-Cox normalization and then sorted. **c** The function returns the top $a_1=5$ normalized variance values in the ordered set $\tilde{{\mathcal {V}}}$ and their corresponding geneset indexes in the ordered set ${\mathcal {I}}$

*Ordering the Genesets* The heuristic idea, which is implemented in function ${ {OrderOfGeneSets}}()$ (Algorithm 1), is visually explained in Fig. 1. In Panel (a), the data matrix *X* has $n=4$ samples (rows) and $p=7$ gene expressions denoted with $f_1, \ldots , f_7$. The geneset matrix *G* is also shown having $m=3$ genesets (rows). For each geneset $g_i$, a **full** PCA is performed restricted to the genes in $g_i$ (Line 6 in Algorithm 1). By full PCA, we mean that all principal components (eigenvectors) and their variances (eigenvalues) are computed. The explained variance for geneset $g_i$ and principal component *j* are denoted with $v_{i,j}$. In Panel (b), the variance values are normalized for geneset size using a Box-Cox normalization (Line 8 in Algorithm 1) and then sorted. The Box-Cox normalized variances are denoted with ${\tilde{v}}_{i,j}$. In the running example, the function returns the top $a_1=5$ normalized variance values in the ordered set $\tilde{{\mathcal {V}}}$ and their corresponding geneset indexes in the ordered set ${\mathcal {I}}$ (Panel (c)).

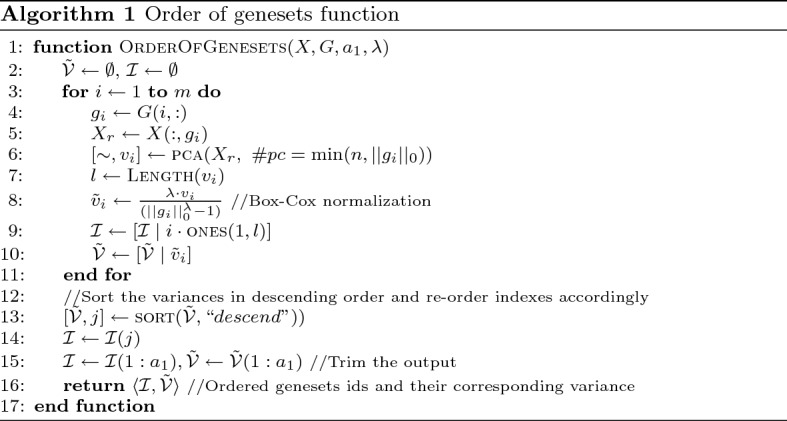



**Fig. 2:**
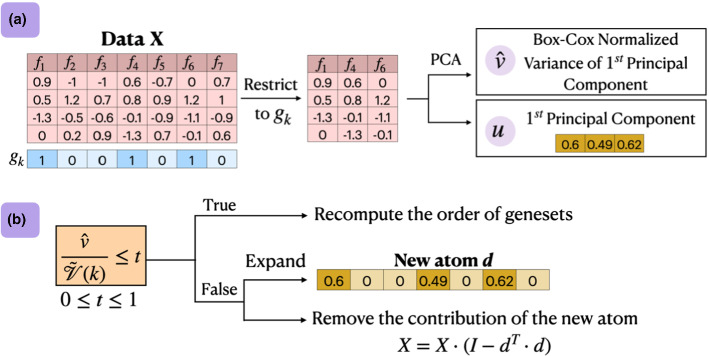
**a** A single PCA restricted to the genes of $g_k$ is performed and the principal component *u* (eigenvector) is obtained. **b** The coefficients for the genes not in $g_k$ are padded with zeros to create the new atom *d*. The contribution of the newly constructed atom is removed from the data. If the actual variance explained by the new atom ${\hat{v}}$ does not match the expected variance $\tilde{{\mathcal {V}}}(k)$, *d* is dropped and the order of genesets is recomputed

*k*-*th (iterative) step*: Given the “next best” geneset $g_k$, PASL constructs the next atom *d* restricted to have non-zeros at the genes of $g_k$ as shown in Fig. 2. A single PCA restricted to the genes of $g_k$ is performed and the first principal component (denoted by *u*) is obtained (Fig. 2a). By single PCA, we mean that only the first principal component and its variance are computed. It corresponds to the non-zero coefficients of *d*. The coefficients for the genes not in $g_k$ are padded with zeros (Fig. 2b). The contribution of the newly constructed atom is removed from the data using $X\leftarrow X\cdot (I-d^T\cdot d)$.

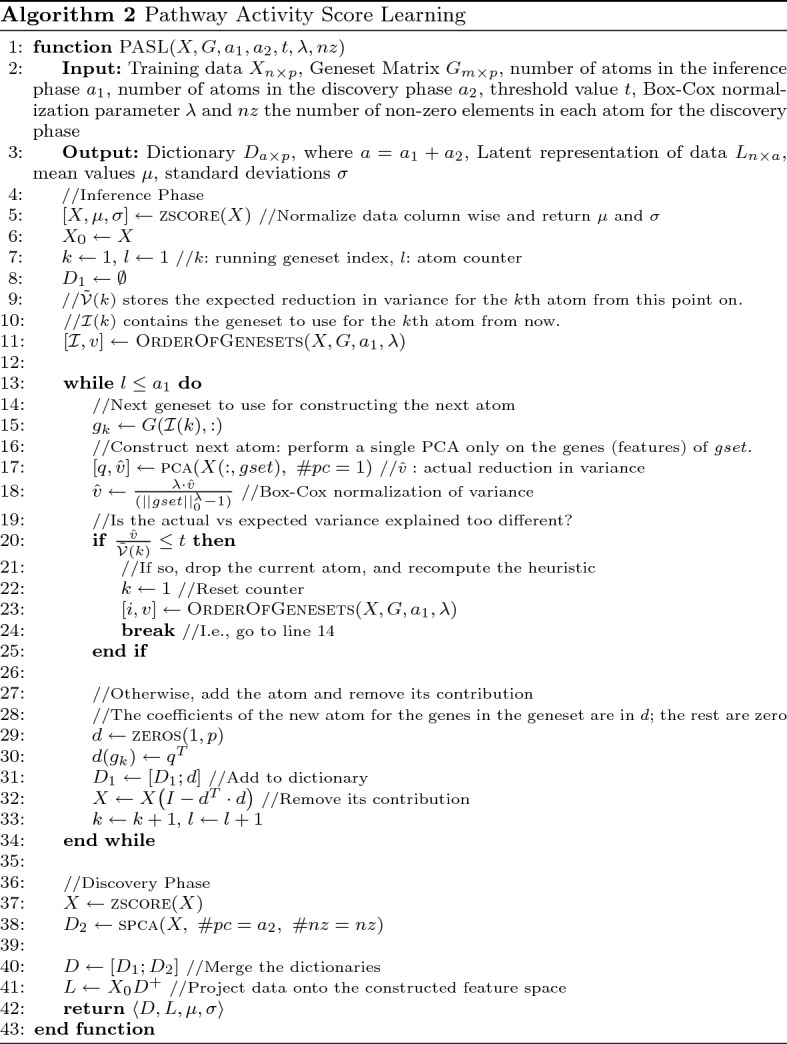



*On explained variance* In Fig. 1c, it is determined to create atoms with an order given by the index set ${\mathcal {I}} = \langle g_1, g_2, g_3, g_2, g_3 \rangle$ in this order. Let us assume that the first atom $d_1$ is created by using the next best geneset ${\mathcal {I}}(1) = g_1$. PASL will then attempt to construct the second atom $d_2$ using ${\mathcal {I}}(2)= g_2$. The ${{OrderOfGenesets}}()$ calculated an **expected reduction** in the (Box-Cox normalized) unexplained variance given by $\tilde{{\mathcal {V}}}(2) = {\tilde{v}}_{2, 1}$ (i.e., the second element in Fig. 1c). However, once the first atom $d_1$ is created, its contribution is removed from the data. Let us call the (Box-Cox normalized) **actual variance reduction** by the current atom $d_2$ as ${\hat{v}}$ (lines 17–18 in Algorithm 2). If $g_1$ and $g_2$ have no intersection (no common genes), then removing the contribution of $d_1$ from the data does not affect the construction of $d_2$. In that case, $\tilde{{\mathcal {V}}}(2) = {\hat{v}}$. Otherwise, $\tilde{{\mathcal {V}}}(2) > {\hat{v}}$, which implies that the actual variance reduction is less than expected.

**Fig. 3:**
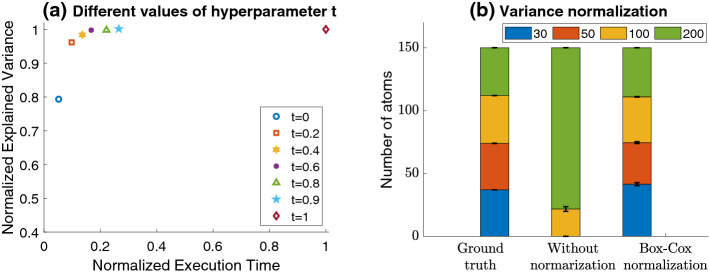
**a** The explained variance (y-axis) as a function of the execution time (x-axis) is shown for different values of *t*. For $0.4 \le t \le 0.9$, the execution time is reduced by a percentage between 65% and 85% with minimal impact on the explained variance. **b** The simulated dictionary (ground truth; left bar) consists of genesets with uniform distribution in terms of sizes to 30, 50, 100, 200 genes. The middle bar shows the distribution of selected pathways when PASL is applied without normalization while the right bar shows the selected pathways when Box-Cox normalization is applied with $\lambda =1/3$. Apparently, the normalization of the variance is necessary for PASL in order to avoid being biased towards selecting genesets with a larger number of genes

*Static vs. Dynamic heuristic strategy* When the discrepancy between the expected and the actual variance explained by the next atom is larger than a threshold, the atom is dropped and the ${{OrderOfGeneSets}}()$ is reevaluated (we refer to lines 20–25 in Algorithm 2 and to Fig. 2b). The specific condition for reevaluation is that the ratio of actual over expected variance explained is smaller than a threshold *t*, or $\frac{{\hat{v}}}{\tilde{{\mathcal {V}}}(2)} \le t$. When $t=0$ the condition always holds. ${{OrderOfGeneSets}}()$ is called just once in the beginning leading to a *static* heuristic strategy. The time complexity of ${{OrderOfGeneSets}}()$ is to perform *m* (the number of genesets) full PCAs. When $t=1$ the ${{OrderOfGeneSets}}()$ will be called with each new atom, selecting the atom that will reduce the variance the most, leading to a *dynamic* heuristic strategy. Its time complexity is to call $a_1$ times the ${{OrderOfGeneSets}}()$. The static strategy is the most time efficient, but sacrifices quality, while the opposite is true for the dynamic strategy. For intermediate values of *t*, small deviations from the optimal order are tolerated and achieve a trade-off between time complexity and learning quality (see Fig. 3a). The dynamic and static strategies coincide when the genesets are mutually exclusive and do not have common genes. The inference phase of PASL is shown in Algorithm 2 (lines 4–34).

*Discovery Phase* The inference phase explains the data variance with atoms that employ genes in known genesets. Unfortunately, not all genes belong in some geneset and not all genesets have been discovered. To capture the remaining data variance, in the discovery phase we create atoms without the restriction that they need to correspond to known genesets. The discovery phase aims to point to new and potentially useful genesets. Based on its generality and efficiency, we employ in our experiments Sparse Principal Component Analysis (SPCA) Zou et al. (2006), Sjöstrand et al. (2012) (line 38 in Algorithm 2). We note though that any other sparse dimensionality reduction technique can be employed. SPCA applies both $l_1$ and $l_2$ penalties in order to regularize and enforce sparsity. We require the SPCA algorithm to return a fixed number of non-zero elements per atom denoted with *nz*. SPCA returns a dictionary $D_2\in {\mathbb {R}}^{a_2\times p}$ and the complete dictionary is the concatenation of $D_1$ and $D_2$, $D = [D_1 ; D_2]$. The discovery phase is at Lines 36–38 of Algorithm 2.

## Setup of evaluation on real gene expression data

We perform two large scale experiments on gene expression data that share a common setup explained below.

*Datasets* All data employed were measured using the Affymetrix Human Genome U133 Plus 2.0 (GPL570 platform) microarray. They were downloaded from the Biodataome repository Lakiotaki et al. (2018). Biodataome^1^ is a collection of uniformly preprocessed datasets, specifically devised for enabling large scale evaluations of data analysis algorithms on biological data. Having the raw measurements uniformly preprocessed makes them comparable across studies and allows them to be pooled together. The datasets are also automatically annotated regarding the disease they pertain to.

*Genesets Employed*: In all experiments the gene membership matrix *G* includes 1974 pathways (genesets) from KEGG Kanehisa and Goto (2000), Reactome Croft et al. (2014) and Biocarta Nishimura (2001), which were downloaded from the Broad Institute Molecular Signatures Database (MSigDB) Subramanian et al. (2005).

*Selection of the Threshold Value* *t* The most time-consuming part of PASL is the execution of the function ${{OrderOfGenesets}}()$ (Algorithm 1) due to the large number of full PCA calculations (one for each geneset). Hyper-parameter *t* controls how often the function ${{OrderOfGenesets}}()$ will be called and the trade-off between learning quality of the dictionary, expressed in percentage of explained variance for a given number of atoms and the execution time. As already presented, it is called at every iteration (every new atom to construct) when $t=1$ while it is called only once at the beginning and never again when $t=0$. In order to determine the optimal value for *t*, we perform an experiment with a merged collection of microarray datasets (Breast Cancer datasets in Sect. 4) where the total number of samples is $n=4235$, the number of genes $p=54675$ and a fixed number of atoms $a_1=200$. Fig. 3(a) demonstrates the explained variance as a function of the execution time for different values of *t*. Based on this plot, *we set t to be equal to 0.9* (cyan star symbol in Fig. 3(a)).

*Selection of the box-cox normalization parameter* $\lambda$ The number of genes, i.e., the number of non-zero elements in each row of the gene membership matrix *G*, varies from few dozens to several thousands. Obviously, the atoms that correspond to larger genesets will have more non-zero coefficients to adjust, therefore those atoms will tend to explain a larger percentage of the variance, everything else being equal. Hence, the larger genesets will tend to dominate the construction of atoms. Indeed, we experimentally observe this phenomenon (see the middle bar of Fig. 3b). Consequently, it is of paramount importance to normalize the variance of each geneset relative to the number of genes it contains. We propose to normalize the variance using the Box-Cox transformation (Box and Cox, 1964) on the number of genes (i.e., on $y=\Vert g\Vert _0$) which is given by

3 $$\begin{aligned} y' = {\left\{ \begin{array}{ll} (y^\lambda - 1)/\lambda &{} \text { if } \lambda \ne 0 \\ \log (y) &{} \text { if } \lambda =0 \end{array}\right. } \end{aligned}$$

where $\lambda$ is a tunable hyper-parameter which controls the power scaling on *y*.

The value of $\lambda$ is determined through a targeted experiment using simulated data generated using genesets with both small and large numbers of genes. Simulated data are generated by first creating a randomly-generated geneset matrix *G* consisting of genesets with sizes uniformly-distributed among the values 30, 50, 100, and 200. Then, we construct a dictionary *D* using randomly-selected genesets from *G*. The non-zero elements of *D* are i.i.d. drawn from uniform distribution $[-1.5,-0.5]\cup [0.5,1.5]$. Finally, $n=400$ samples with $p=500$ features are generated from *D* and the activation scores matrix *L*. In order to avoid spurious effects from scores close to zero, the elements of *L* are uniformly distributed in $[-1.5,-0.5]\cup [0.5,1.5]$.

After extensive tests with a wide range of values for the Box-Cox transformation hyper-parameter, we set $\lambda = 1/3$. The geneset selection results obtained with PASL are presented in Fig. 3(b). Evidently, *the use of Box-Cox transformation with* $\lambda =1/3$ *(right bar) produced results similar to the ground truth (left bar) while PASL without normalization failed to correctly infer the true dictionary (middle bar)*.

*Remaining Hyper-parameter Values* We set the number of non-zero elements at the discovery phase (i.e., *nz* in SPCA) to be equal to 2000. However, we have to mention that we do not tune this hyper-parameter due to the lack of a clear criterion to optimize for. Finally, the number of atoms in the dictionary is specified in each experiment separately.

*Construction of the PASL Latent Feature Spaces* When samples are split between training $X_{train}$ and test $X_{test}$ sets, ${PASL}({X_{train}, G, a_1, a_2, 0.9, 1/3, 2000})$ (Algorithm 2) is called with input $X_{train}$ to obtain a dictionary *D*, mean values $\mu _{train}$, and standard deviations $\sigma _{train}$.

Then, ${TransformByPASL}({X_{test}, \mu _{train}, \sigma _{train}})$ (Algorithm 3) transforms the $X_{test}$ data to a latent representation $L_{test}$. The first $a_1$ columns of $L_{test}$ correspond to pathway activation scores for known genesets and the remaining $a_2$ columns to scores of discovered genesets. The use of $\mu _{train}$ and $\sigma _{train}$ to standardize test data ensures that the test data do not bias their transformation and the estimation of metrics such as reconstruction error or predictive performance. It also implies that no quantities are estimated from the test data to transform them, hence one could transform even a single test sample.

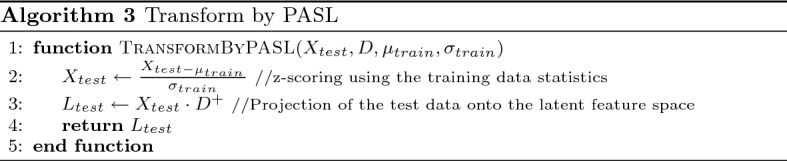



**Fig. 4:**
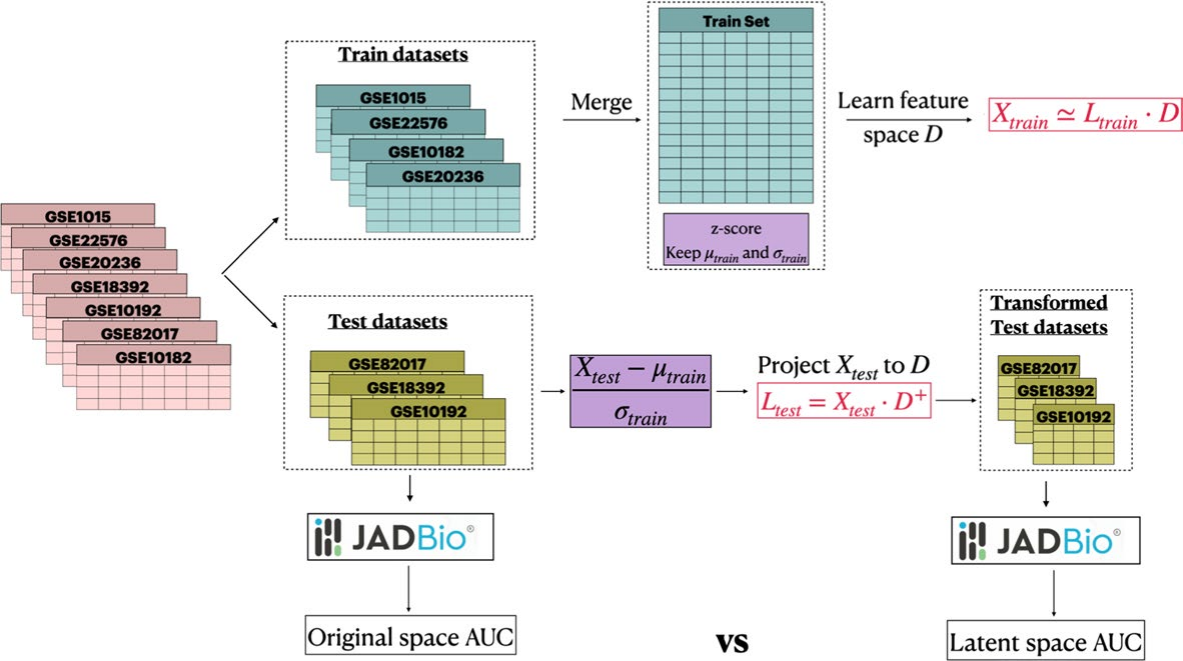
Evaluation protocol. The datasets are split into train and test datasets. The train datasets are merged creating a large dataset. PASL is applied on the train set and the final evaluation in terms of predictive performance is performed on new test datasets. The initial test datasets are compared against the lower-dimensional transformed datasets in terms of predictive performance

*AutoML analysis with JADBio:* We used JADBio for all predictive modelling tasks (version 1.1.21, www.jadbio.com). JADBio has been developed specifically for small-sample, high-dimensional data, such as multi-omics data. JADBio uses the SES (Lagani et al., 2016) and LASSO feature selection algorithms, combined with ridge logistic regression, decision trees, random forests, and SVMs for modelling. It automatically tries thousands of combinations of algorithms, simultaneously tuning their hyper-parameters. The exact hyper-parameter values tried depend on the size and type of the data and are determined by JADBio’s AI system. JADBio outputs include the final winning model produced by the best configuration (pipeline of algorithms and hyper-parameter values), the set(s) of features selected within the winning model, and a conservative estimate of the model’s performance based on the BBC-CV protocol (Tsamardinos et al., 2018). The latter is a version of cross-validation that adjusts performance estimates of the winning model for trying multiple configurations. Without this adjustment, the cross-validation performance of the winning combination is optimistic. A detailed description of the platform along with a massive evaluation on hundreds of omics datasets is included in Tsamardinos et al. (2020). JADBio has produced novel scientific results in nanomaterial prediction (Tsamardinos et al., 2020), suicide prediction (Adamou et al., 2018) and others. The use of JADBio is meant to ensure that (a) performance estimates are accurate, and (b) results do not depend on a single ML algorithm tried with just the default hyper-parameters. Predictive performances are reported in terms of Receiver Operating Characteristic Area Under the Curve (AUC), a metric that is insensitive to the proportions between different classes and that has a simple interpretation, with 1 representing perfect classification, 0.5 random guessing and 0 perfectly inverted classification. For multi-class problems, an AUC is constructed for each class serving as the “Positive" class. The reported AUC is the average over all classes. A visualization of the experimental setup and evaluation protocol is shown in Fig. 4.

## Comparing PASL against PLIER on breast cancer and leukemia studies

For this first set of experiments we downloaded all the available (as of May 2020) datasets in Biodataome related to Breast cancer and Leukemia, each having at least 20 samples. The datasets form the Breast Cancer collection and Leukemia collection. For each collection we select 80% of the datasets to pool together and use them as training data. The remaining 20% of the available datasets are employed as test dataset and are *not seen by any dimensionality reduction method during training*. Hence, samples from the same dataset either belong in the train data or the test data but not both. The selection of datasets used for the train or the test set is random, with the restriction that test datasets have to be accompanied by a discrete binary or multi-class outcome. The outcomes are clinically interesting quantities provided in the original studies, such as the disease status, the response (rapid/slow) to a specific treatment,mutation status, hormone status, dietary restrictions etc. *The annotation of each sample with an outcome was performed manually by the authors*. The training set for the Breast cancer and the Leukemia collection contains 4235 and 5694 unique gene-expression profiles, respectively. For this set of experiments, the geneset matrix *G* was built by using the annotation provided by the vendor of the microarray chip^2^.

We comparatively evaluate PASL against a recently introduced algorithm called PLIER (Mao et al., 2019). Like PASL, PLIER learns a latent feature space that corresponds to known genesets. PLIER also accepts as input data *X* and a geneset matrix *G*. Similarly to PASL, it returns the scores *L* and the dictionary *D*, such that $X \approx L \cdot D$. PLIER accepts several hyper-parameters. The maxpath hyper-parameter indicates how many genesets an atom of *D* is supposed to correspond to. We set maxpath = 1 requesting that each atom in *D* corresponds to one and only geneset, so that the output is comparable to PASL. Unfortunately, *PLIER treats maxpath as indicative; atoms in D may correspond to the union of several genesets, even when maxpath = 1*. In that sense, the atoms in *D* are not as easy to interpret as the ones returned by PASL. PLIER also ignores genesets with fewer features than minGenes. We set minGenes = 1 so that no genesets are ignored. Finally, we note that in PLIER the scores *L* are computed as $X\cdot D^T \cdot (DD^T+\lambda _2 I)^{-1}$, where $\lambda _2$ is a regularization parameter learned by the algorithm.

PASL and PLIER dimensionality reduction are both applied on the training sets of both Breast Cancer and Leukemia collections and learn the dictionaries of atoms. The atoms of PLIER are not as sparse as the ones output by PASL. For example, for the Breast Cancer collection analysis, the mean number of non-zero coefficients in each atom of PLIER is 25833 (almost half of the original feature size), while for PASL it is 1329. For the same number of atoms, PLIER uses more degrees of freedom (non-zero coefficients) to find a suitable transformation to a latent space. For a fair comparison in the subsequent experiments, we impose the restriction that the learned dictionaries $D_{\text{ P }LIER}$ and $D_{\text{ P }ASL}$ have approximately the same number of non-zero elements. To this end, we first run PLIER allowing it to construct a large number of atoms and estimate the number of atoms *a* required to reach approximately the same number of non-zeros as PASL. Then, we re-run PLIER constrained to produce only *a* atoms. Specifically, when PASL is restricted to 500 atoms, its dictionary contains 664965 and 700020 non-zeros for the Breast Cancer and the Leukemia collections, respectively. PLIER is limited to 29 and 30 atoms instead, producing dictionaries with 699976 and 782114 non-zeros, respectively. The $D_{\text{ P }LIER}$ and $D_{\text{ P }ASL}$ were then used for projecting each test set on their respective latent feature spaces.

### Predictive performance in latent feature space

**Table 2:** AUC of the test datasets for PASL, PLIER and Original space (initial test datasets). PASL and PLIER are tested for approximately equal number of non-zero entries in the dictionary matrix. For Breast cancer data PASL’s latent space consists of 500 dimensions-664965 non-zeros. PLIER’s latent space consists of 29 dimensions of 699976 non-zeros. For Leukemia, PASL’s latent space consists of 500 dimensions of 700020 non-zeros. PLIER’s latent space consists of 30 dimensions of 782114 non-zeros

Breast Cancer				Leukemia			
Data ID	PASL	PLIER	Original	Data ID	PASL	PLIER	Original
54002	0.999	1	0.995	15434	0.985	0.747	0.987
5460	0.952	0.958	0.96	14924	0.996	0.987	0.91
36771	0.935	0.933	0.963	23025	0.762	0.766	0.741
66161	0.664	0.486	0.579	21029	0.95	0.694	0.966
76124	0.976	0.98	0.97	28654	0.767	0.616	0.762
66159	0.759	0.506	0.776	14671	0.59	0.674	0.625
66305	0.513	0.569	0.535	7440	0.73	0.52	0.736
10780	0.976	0.995	0.962	66006	0.926	0.792	0.952
27562	0.835	0.776	0.914	28460	0.719	0.542	0.697
27830	0.725	0.671	0.759	26713	0.998	0.997	0.952
36769	0.953	0.963	0.96	31048	0.984	0.981	0.99
29431	0.997	0.982	0.991	39411	0.997	0.956	0.985
42568	0.991	0.975	0.927	49695	1	0.612	0.998
				50006	0.979	0.994	0.983
				61804	0.823	0.744	0.869
**Mean**	**0.8673**	** 0.830**	**0.868**	**Mean**	**0.8804**	**0.7748**	**0.876**
**Median**	**0.952**	**0.958**	**0.96**	**Median**	**0.95**	**0.747**	**0.952**

The values in bold are the mean and median AUC for the previous rows of each different case (PASL, PLIER, Original Space)

**Fig. 5:**
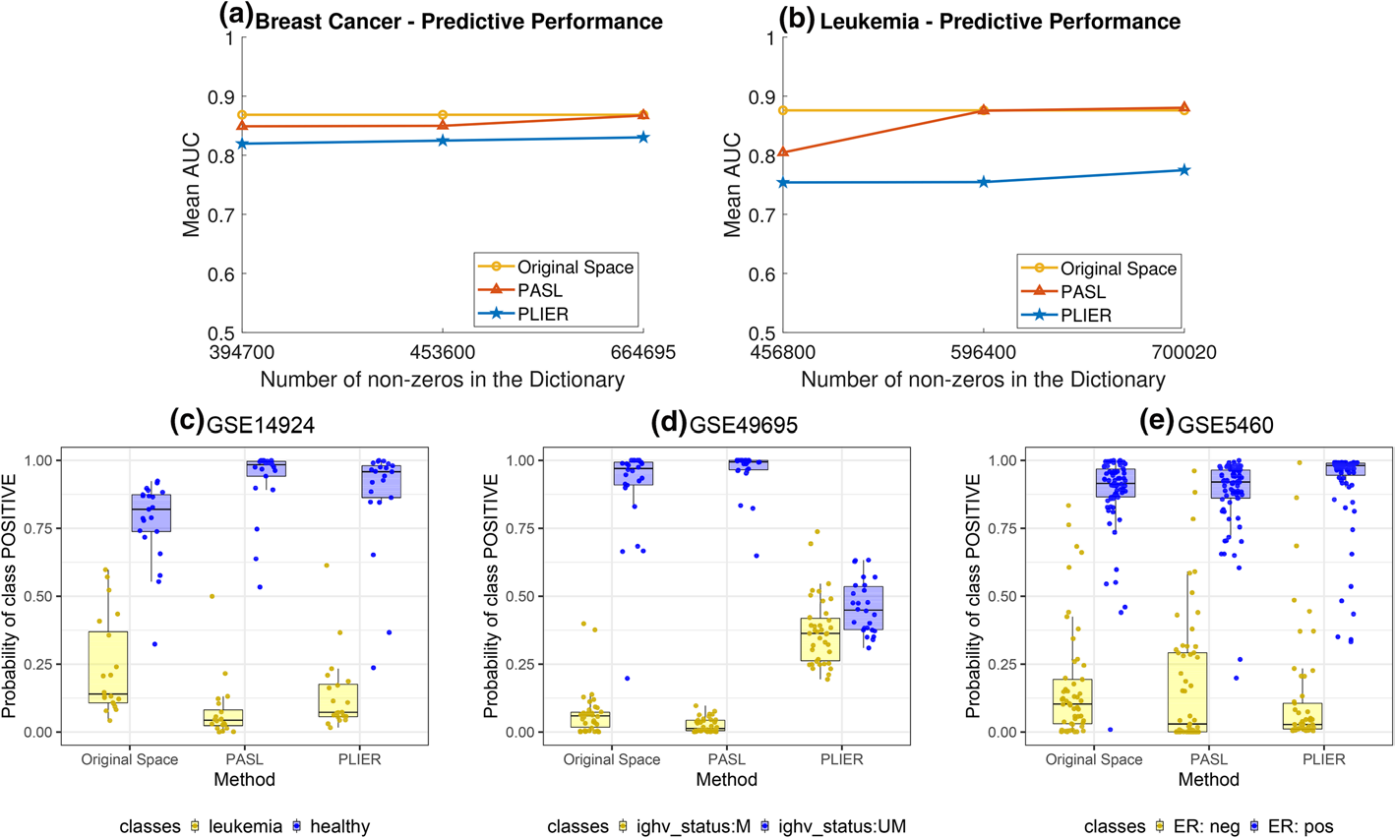
**Upper row** Mean AUC of **a** Breast Cancer and **b** Leukemia test datasets **Lower row:** *Out-of-sample probability distributions* (calculated during cross-validation) of the predicted probabilities by JADBio for different datasets and data representations (original, PASL, PLIER). We visualize the datataset that favors the most a specific algorithm. The larger the separation of the predicted probabilities for each class, the better AUC is achieved by the predictive model. **c** dataset favoring PASL over original representation (outcome is disease status), **d** dataset favoring PASL over PLIER (outcome is the mutation status of immunoglobulin heavy chain (IGHV) gene), **e** dataset favoring PLIER over PASL (outcome is the status of the ER receptor)

We performed classification analysis using JADBio on 13 and 15 test datasets for Breast Cancer and Leukemia, respectively. The analysis uses the original feature space, as well as the PLIER and PASL feature spaces, for different dimensionalities. For PASL, the number of atoms to learn take the values 250, 400, and 500. The number of atoms with approximately the same number of non-zeros in the dictionary of PLIER is 20, 25, and 30. Thus, there are 7 analyses for each dataset, and $91+105$ analyses in total. *For the Breast Cancer (resp., Leukemia) datasets 860002 (resp., 983425) classification models were trained in total by JADBio with different combinations of algorithms and hyper-parameter values on different subsets of the input data (cross-validation).*

Regarding execution time, the analysis in the space of PASL or PLIER takes about *1 order of magnitude less time* than in the original space. The exact execution time in JADBio depends on several factors, such as the load of the Amazon servers on which the platform runs, and thus exact timing results are meaningless. Indicatively, we mention a typical case: the analysis of GSE61804 for the original space took 1.15 hour, while it took 9 minutes and 5 minutes for PASL and PLIER, respectively. Figure 5a and b show the average AUC over all test datasets for each disease for increasing number of non-zeros. *PASL outperforms PLIER and it is on par with analyses on the original space*. Thus, the learned dictionary by PASL generalizes to new test data and captures the important information to perform classification with various disease-related outcomes. At the same time, *PASL achieves 2-orders of magnitude dimensionality reduction by a sparse matrix whose atoms directly correspond to known genesets (pathways)*.

We now focus on the experiments for the largest dimension of PASL and PLIER. The number of atoms in PASL is set to 500 (664965 non-zeros for Breast Cancer, 700020 non-zeros for Leukemia). PLIER’s latent space consists of 29 (699976 non zeros) and 30 (782114 non-zeros) atoms for Breast Cancer and Leukemia respectively. Table 2 contains the detailed results for each dataset and method. The worst case (best case) for PASL is dataset with ID 27562 (14924) where it achieves 8 AUC points (8 AUC points) lower (higher) performance vs no dimensionality reduction. In contrast, there are several datasets (IDs 66161, 66159, 27562, 15434, 21029, 7440, 66006, 28460, 28460, 49695, 61804) where PLIER’s performance is lower than 10 or more AUC points.

In the lower row of Fig. 5 we visually demonstrate the ability of PASL to lead to highly predictive models. Each panel corresponds to a different test dataset. Specifically, we chose to present the visualizations from datasets that lead to the “best” visual differences for PASL vs the original space, PASL vs PLIER, and PLIER vs PASL, in Fig. 5(c)–(e), respectively. Each panel shows the box-plots of the *out-of-sample probability* of each molecular profile to belong to the positive class for the models produced in the original, PASL, and PLIER feature space. The out-of-sample predictions are calculated by JADBio during the cross-validation of the winning configuration (ML pipeline) and thus, they do not contribute to the fitting of the samples used for training. The larger the separation of the distribution of the predicted probabilities, the larger the AUC.

### From gene set enrichment analysis to differential activation analysis

**Fig. 6:**
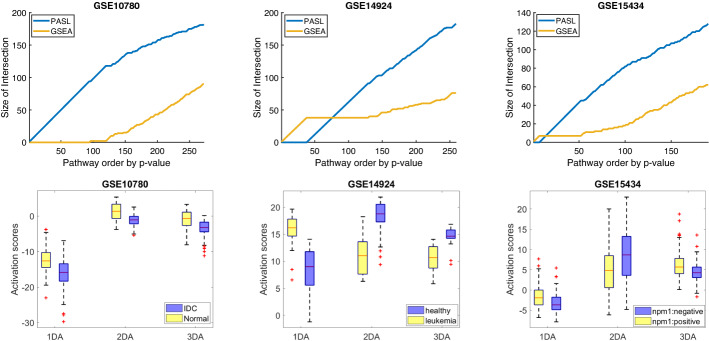
**Upper row:** Comparing DAA (differential activation analysis in PASL space) and GSEA (standard geneset enrichment analysis). For a given *x* (e.g. 100) in the x-axis, and for the blue line corresponding to PASL, the value *y* (e.g., 95) in the y-axis is the number of genesets within the top *x* most statistically significant genesets that are identified by PASL’s DAA. Similarly, for the orange line for GSEA. The genesets identified with DAA in PASL space have in general smaller *p*-values (higher statistical power) and are encountered first in the sorted list of genesets. DAA and GSEA are complementary in identifying genesets that are differently affected in two experimental conditions. **Lower row:** Box-plots of the activation scores that correspond to the first, second, third differentially activated PASL feature/pathway denoted by 1DA, 2DA, and 3DA, respectively. It is visually verified that the differentially activated pathways indeed behave differently between the two classes. The outcomes for GSE10780, GSE14924, and GSE15434 stands for Invasive Ductal Carcinoma/Unremarkable breast ducts, healthy-controls/leukemia-cases, the mutation status of Nucleophosmin 1 (NPM1), respectively (Color figure online)

We performed a computational experiment assessing the biological interpretability of PASL’s feature space. Since the constructed features correspond to known or discovered genesets (atoms of *D*), we can use their values (stored in the columns of *L*) to find which genesets behave differently under two conditions, i.e., disease vs. healthy or treatment vs. control. In other words, we can perform Differential Activation Analysis (DAA). DAA is to genesets what standard differential expression analysis is to genes. A current standard alternative method that provides insight into the underlying biology is to use Gene Set Enrichment Analysis (GSEA). GSEA first summarizes the probe-sets that correspond to the same gene e.g. by taking the minimum, maximum or average expression value. Inherently, GSEA loses information by applying this summarization and by not taking into account the covariances of the gene expressions. Subsequently, the null hypothesis is that the *p*-values of the genes in a pathway have the same distribution as the *p*-values of the genes that do not belong to the pathway.

We next examine the ability of PASL to identify genesets (pathways) that behave differently between two classes and compare it against GSEA. We employ the GSEA v4.0.3 tool from https://www.gsea-msigdb.org/gsea/index.jsp Mootha et al. (2003), Subramanian et al. (2005). We run GSEA on the test datasets in the original feature space using 10000 phenotype permutations for the permutation-based statistical test employed in the package. The input genesets are the same as the ones provided to PASL in the geneset matrix *G*. We also perform DAA on the test datasets projected to the latent space of PASL (activity scores) using the Matlab’s t-test function *mattest* with 10000 permutations. The list of *p*-values from DAA and GSEA can then be used to identify the affected pathways.

Figure 6 (upper row) shows the number of genesets identified by each method (y-axis) in the top *k* (lowest *p*-value) genesets, for each *k* (x-axis). Each panel corresponds to a different test dataset. We observe that the genesets identified by PASL have lower *p*-values and are encountered first on the list; PASL has higher statistical power in identifying some genesets that behave differently. PASL’s features correspond to genesets. The statistically significant ones are referred as *differentially activated*. Figure 6 (bottom row) visualizes why the PASL features are identified as *differentially activated*. Each panel shows the box-plots for the activation scores corresponding to the first, second, and third most statistically significant PASL feature/geneset (denoted with names 1DA, 2DA, and 3DA, respectively).

Specifically, the top 3 differentially activated genesets of GSE10780 are the “Reactome signaling by GPCR", “Reactome Fructoce Catabolism" and “Reactome Hemostasis". The top 3 differentially activated genesets of GSE14924 is the “Reactome metabolism of Lipids", “Reactome Chromatin Organization" and “Reactome Gene Expression Transcription". The top 3 differentially genesets pathways of GSE15434 are the “Reactome Transport of Small Molecules", “Reactome Developmental Biology", “Reactome Post Translational Protein Modification". *It is visually verified that the distribution of the activation scores of these genesets are different between the two classes in an easy to understand and intuitive plot.*

While DAA using PASL seems to offer several advantages (lower *p*-values, intuitive visualization), it also has a major limitation. PASL requires a training set that is related to the application (test) set. It learns atoms that only pertain to capturing information regarding the train data. We consider DAA and GSEA complementary and synergistic since, for instance, DAA using PASL cannot be applied to a schizophrenia dataset, before we construct a sufficiently large training dataset for the disease. To alleviate this data-stemmed limitation, a full-scale experiment where datasets from a wide range of diseases and pathologies are merged is presented next.

## Construction of a universal dictionary for the GPL570 gene expression platform

In this experiment we aim to build a universal dictionary for the Affymetrix Human Genome U133 Plus 2.0 platform (GPL570). “Universal” in this context means that the latent representation should be (a) applicable to any dataset measured through the GPL570 platform, and (b) able to retain all relevant information from the 54675 original probe-sets in a much lower dimensional space. Such representation would enable faster computational analysis and enhanced interpretability of the results.

**Fig. 7:**
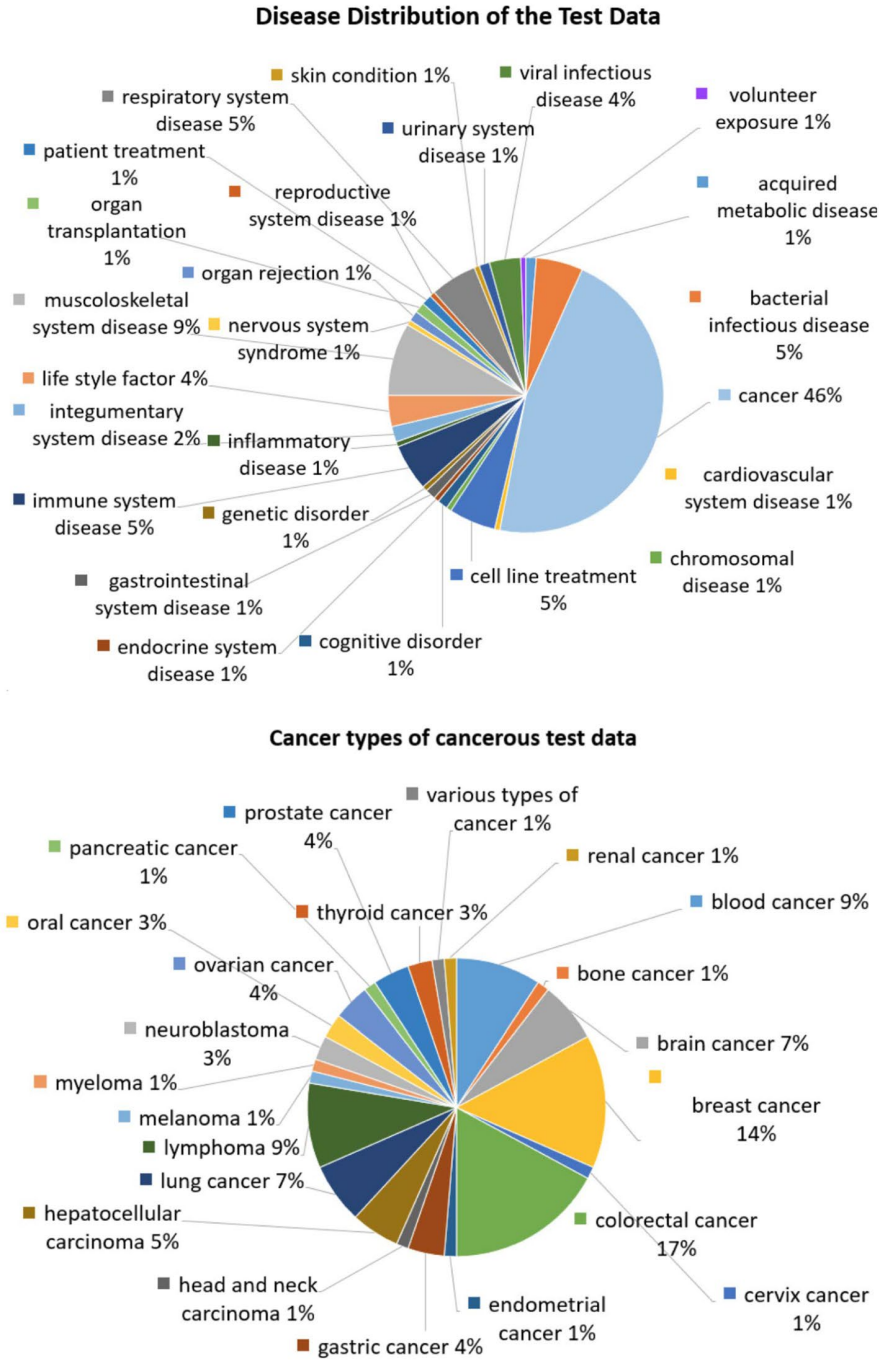
**Top:** Disease distribution of the test datasets. The test datasets refer to 25 different diseases. **Bottom:** Distribution of the cancer types along the cancerous test datasets. The cancerous test datasets refer to 22 different types of cancer

We downloaded all available GPL570 datasets with at least 20 samples, for a total of 1736 datasets. We randomly selected 173 (10%) of these studies to hold out for testing purposes, *leaving 1563 datasets with 85643 samples for dictionary training and validation*. Out of the 173 tests datasets, 8 were discarded for not having a suitable binary or multi-class outcome, leaving a total of *165 test datasets with a total of 16286 samples*. On the dataset level, we manually annotated each dataset for the studying purpose of the corresponding project and the disease it pertains to. On the sample level, we manually labeled each sample with every possible information available such as disease, treatment, sex, ethnicity etc. We then chose to define as a classification outcome the most biologically important quantity in the study. In the top pie chart of Fig. 7 we show the distribution of the types of diseases of the test datasets. Specifically the test datasets refer to 25 different disease types. 46% of them refer to 22 different types of cancer, whose distribution is shown in the bottom pie chart of Fig. 7. The gene membership matrix *G* reporting the correspondance between probe-sets and genesets was built using the GSEA tool (https://www.gsea-msigdb.org/gsea/index.jsp).

We note that the construction of a universal dimensionality reduction transformation has been presented by the authors in prior work (Pantazis et al., 2020). In the latter, the authors apply PCA, Kernel PCA and Autoencoders on a large corpus of microarray and RNAseq data. The projection to the estimated universal dictionary outperforms the original space in terms of predictive information. However, all methods tried lead to biologically uninterepretable transformations.

### Learning a universal dictionary with PASL on large sample datasets

Applying PASL on 85643 samples presented numerous technical challenges that had to be overcome. The dataset does not fit in main memory; intermediate calculations also require memory that reduces the total number of samples that can be loaded. Loading only portions of the data for the different PCAs performed by the algorithm is an option to reduce memory requirements, but it results in excessive I/O of data.

We performed numerous optimizations to PASL to reduce memory requirements. First, the data were represented with single instead of double precision, reducing memory requirements to half. Extensive experiments with single precision on sub-samples verified that there is no loss of explained variance by the learned dictionary (not shown for brevity). PASL computations that are memory hungry were also optimized; surprisingly, this includes the *zscore* built-in function of matlab that does not run with such large datasets. The dictionary *D* is now represented in a sparser form to reduce memory requirements. After these optimizations in place, we managed to run the inference phase of PASL with a randomly chosen sub-sample of 50000 samples to learn 5000 atoms, and the discovery phase (Sparse PCA) with 15000 samples to learn 1000 atoms with 2000 non-zero elements each. The two phases required about 3 days to run. The computer that we used to run PASL has the following specs: CPU: Intel Core i7-8700K @ 3.7GHz (6 Cores / 12 Threads), RAM: 32GB DDR4-2666MHz. The experiments ran on Matlab 2017a.

### Description of the constructed dictionary

**Fig. 8:**
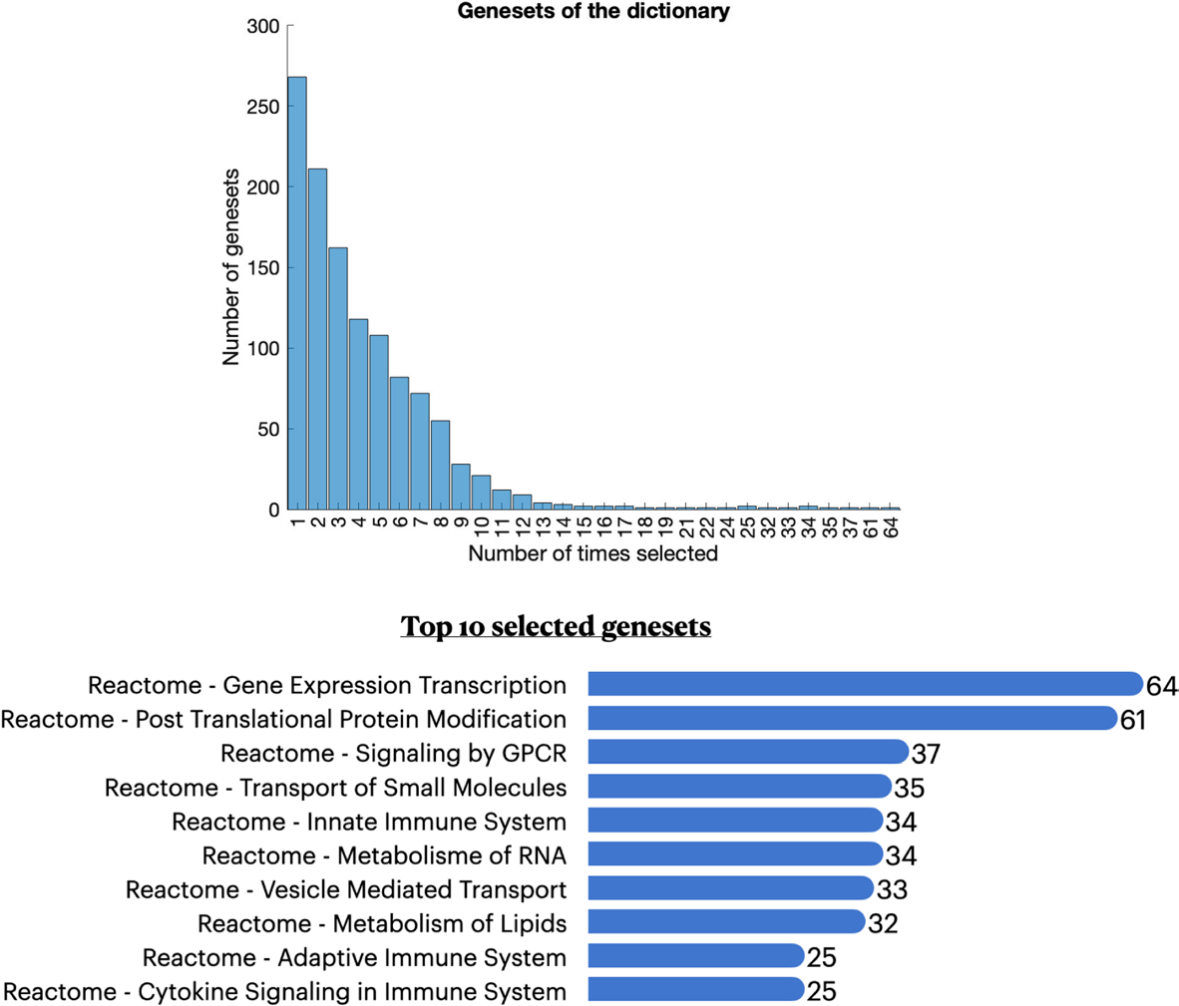
**Top:** Distribution of the number of atoms that correspond to the same geneset. Most genesets correspond to a single atom in the dictionary. **Bottom:** 10 most frequently selected genesets of the dictionary. A geneset that corresponds to several atoms behaves differently under different conditions. This explains why these genesets correspond to general and fundamental biological pathways

The 5000 atoms of the inference phase correspond to 1174 unique known genesets out of the available 1974. In the top panel of Fig. 8 we show the distribution of the number of atoms that correspond to the same geneset, i.e., how many times a geneset was selected to create an atom. More than 250 genesets correspond to a single atom. In the bottom panel of Fig. 8 we present the top 10 selected genesets and the number of atoms that correspond to them. For a geneset to correspond to numerous atoms, it means that its behavior cannot be captured with a single score. The genes that participate in such a geneset could correlate differently depending on the biological context (treatment condition, pathology, tissue). This observation explains why the genesets with the largest number of atoms are quite general. The universality of PASL GPL570 latent representation lies in the large number of included genesets and their diversity. The dictionary includes atoms from a wide spectrum of genesets that correspond to both foundational biological processes, such as Gene Expression Transcription (Fig. 8), as well as more specific biological pathways that are currently associated only with a handful of diseases, such as “RUNX3 regulates CDKN1A transcription”, “NTRK2 activates RAC1” and “ATF6 (ATF6-alpha) activates chaperone genes”. This variety of included genesets enables the GPL570 PASL dictionary to represent datasets corresponding to different diseases with high precision.

### Predictive performance of the universal dictionary

**Table 3:** Mean and median AUC achieved on the 165 test datasets by JADBio in original gene expression and PASL space, respectively. The mean and median execution time is also reported. There is no loss of predictive performance on average across all tissues, pathologies, and outcomes in the test sets; predictive analysis is sped up by about a factor of 5 on average

	Mean	Median	Mean	Median
	AUC	AUC	Exec. time	Exec. time
PASL space	0.914	0.972	8800 sec	3833 sec
Original space	0.916	0.969	45962 sec	22791 sec

**Fig. 9:**
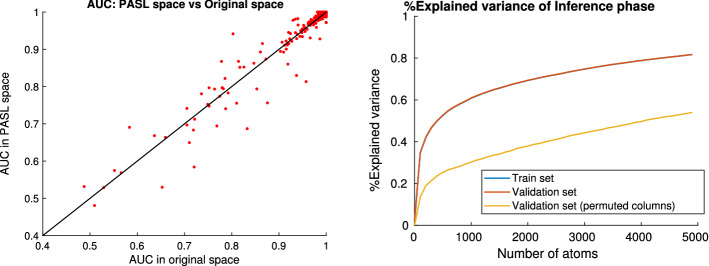
**Left:** Out-of-sample AUC estimates of JADBio models achieved in PASL and the original gene expression space for the 165 test datasets. The estimates correlation is 0.95 . Some datasets are better predicted in PASL space (points above the diagonal), while the opposite is true for the points below the diagonal. **Right:** Percentage of explained variance of the inference phase; only genes that belong in at least one geneset are included. By permuting the columns (features) in the validation set we get the explained variance by a random sub-space projection with the same sparsity level as PASL. PASL’s explained variance on the training and validation sets coincide showing no overfitting of the dictionary. The explained variance is significantly higher than the random projection

In Table 3 we present the mean and median AUC for the out-of-sample estimate of the predictive performance achieved by JADBio models for the original and the PASL feature space. In total, JADBio created 18,576,046 classification models in its effort to identify the configuration that leads to the most predictive model and to estimate its performance (cross-validation). All JADBio analyses on the 165 test datasets were performed on the ARIS high-performance computing system provided by the national Greek GRNET initiative. In the left panel of Fig. 9 we present a per-dataset comparison of predictive AUC in the PASL and the original gene expression space. Each point represents a test dataset. Points above the diagonal achieve higher predictive performance in PASL space than the original space and vice versa. As the figure suggests, there is a high correlation (0.95) between the AUC values obtained using the original and the PASL features. Furthermore, no statistically significance difference exist between the means of two sets of AUC values (paired t-test p-value: 0.54), while the execution time is significantly smaller with the constructed features (*p* value:2.7e-8). The results in Table 3 and Fig. 9 (left panel) indicate that the transformed space is indeed able to retain all information needed for predicting relevant outcomes contained in the original features, and that predictive analyses can be performed more efficiently in the lower dimensional space.

Since only 50000 samples out of the 85643 total available samples were used to train the dictionary, we used the remaining samples as a *validation set*. In the right panel of Fig. 9 we present the percentage of explained variance of the train set, the validation set and the validation set with a random permutation of the columns (features). The percentage of explained variance is computed by the following formula:

4 $$\begin{aligned}  {\% \,\text{Explained}\, \text{Variance} } = 1 - \frac{|| X_z - L \cdot D_1 ||_F^2}{ || X_z ||_F^2} \end{aligned}$$

where $X_z$ is the standardized data matrix, $D_1$ is the dictionary of the inference phase and *L* is the projection of the data to the dictionary $D_1$. We note that *we have removed the genes that do not appear in any geneset for this calculation* to focus on the part of the variance that is feasible to explain with the $D_1$ dictionary. By permuting the features in the validation set, we compare the explained variance achieved by $D_1$ with a random projection of the data to a sub-space of the same sparsity level as $D_1$. We note that *(a) the variance explained by* $D_1$ *on the validation data coincides with the variance explained on the training data*; the two lines fall onto each other and are indistinguishable in the figure. *(b) the variance explained by* $D_1$ *exceeds that of random projections.* However, notice that a random projection to a subspace still captures a portion of the variance. This behavior is consistent with linear projection theory since a random projection given sufficiently many atoms is capable of maintaining the distance between the samples in the projected space and thus be capable of reconstructing the samples when mapped back to the original space. More specifically, Johnson–Lindenstrauss lemma Johnson and Lindenstrauss (1984) states that a set of points in a high-dimensional space can be linearly embedded into a space of much lower dimension in such a way that distances between the points are nearly preserved. The relative error is controlled by the number of atoms and samples but not by the samples’ dimension. Moreover, the random projection’s relative error is on average of order $1-\varOmega \left( \sqrt{8\log (n)/a_1}\right)$ which qualitatively is very similar with the percentage of explained variance in the right panel of Fig. 9.

## Illustrative case-studies of predictive modeling with JADBio in PASL space

Out of the 165 test datasets that were analysed by JADBio, we select to focus on two of them that illustrate the potential advantages of predictive modeling in PASL space. More details about the datasets and the JADBio analyses can be found in Sects.  6.1 and 6.2. JADBio can share results to the community in interactive web pages with unique urls. The urls to interactively explore the results are in the respective subsections.

### Classification of Acute Lymphoblastic Leukemia (ALL) patients with and without Down Syndrome

The work in Loudin et al. (2011) is a study of the differences between biological mechanisms in patients with pediatric Acute Lymphoblastic Leukemia (ALL) that have Down Syndrome (DS-ALL) against ALL patients without Down syndrome (NDS-ALL). This defines a binary outcome and corresponding classification problem. To our understanding, the original study attempted to find a diagnostic signature but failed. Specifically, they report “As expected, unsupervised hierarchical clustering analysis demonstrated clustering of NDS-ALL cases belonging to some known cytogenetic subgroups such as E2A-PBX1 and MLL rearrangement (Fig. 3a). In contrast, neither DS-ALL cases overall nor the JAK2-mutated, histone deletion or high CRLF2 expressing DS-ALL cases formed a cohesive cluster." Given that they DS-ALL cases did not cluster, they proceed with supervised analysis of other outcomes.

**Fig. 10:**
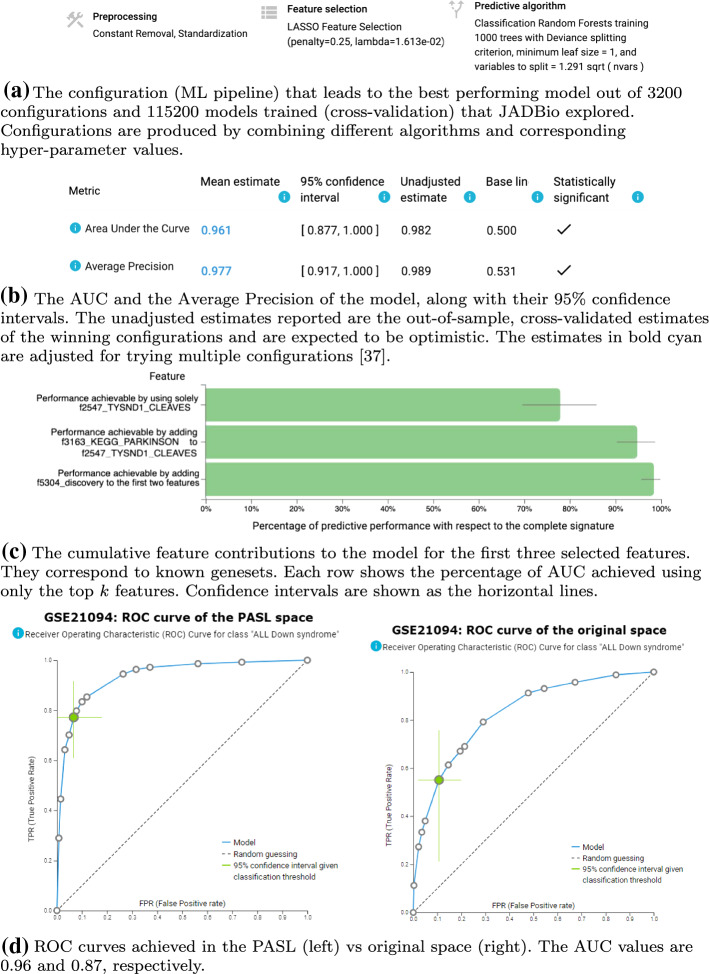
Predictive modeling with JADBio of Down Syndrome vs Non Down Syndrome in ALL cases (dataset GSE21094). Panels a, b and c (left) were produced in the PASL space (https://app.jadbio.com/share/81be792e-3ba8-43e0-ace0-b8a4fe9657f0), panel c (right) in the original space (https://app.jadbio.com/share/32892dc1-739d-4b37-b6b5-50802587fb1b)

The data are reported in dataset GSE21094 contain 49 total samples, 23 DS-ALL and 26 NDS-ALL. The analysis in the original space led to a single signature^3^ (a signature is defined as the subset of selected features) with 25 mRNA predictors, achieving an AUC of 0.865 (0.778, 0.945) using a Support Vector Machine as best model. The analysis in the PASL space led to a Random Forest type of model (Fig. 10a and a sizable increase in predictive performance with an AUC 0.961 (0.877, 1.000) (Fig. 10b. There was a single signature containing 23 features. Figure 10c shows the cumulative contribution of the first 3 selected features, i.e., the percentage of AUC achieved when features are added one at a time. Fig. 10d shows the ROC curves of the PASL space (left panel) and the Original space (right panel).

Regarding the biological interpretation of the selected PASL features, the top two directly correspond to the genesets “Reactome TYSND1 cleaves peroxisomal proteins” and “KEGG parkinsons disease”. The third feature originates from PASL’s discovery phase. It does not have a direct match to a known pathway, but could be pointing to a novel pathway. The TYSND1 gene encodes a protease that removes the N-terminal peroxisomal targeting signal from proteins PTS2 in the cytosol and facilitates their import into the peroxisome. In the peroxisomal matrix, it removes the C-terminal peroxisomal targeting signal PTS1 which leads to peptide degradation Kurochkin et al. (2007). Peroxisomal defiency is common in Zellweger Syndrome, a Down syndrome’s mimic (Khadpe et al., 2019) and in a variety of neurological diseases such as Alzheimer’s, Parkinson’s disease and Down syndrome (Berger et al., 2016). From a clinical perspective, discovering a geneset related to Parkinson’s disease as predictor is expected in this context, as early-Onset Parkinsonism is prevalent in Down Syndrome (Hestnes et al., 1997). The 25 mRNA expression in the signature in the original space encode a variety of proteins with different biological roles such as binding membrane protein, proteins involved in cilium biogenesis and small nuclear RNA factor. Interestingly, one of them, Ephrin type-A receptor 7, is involved in the brain development pathway. Arguably however, it is easier to interpret biologically the PASL features that correspond to pathways, than the individual gene expressions. The JADBio analyses both for the PASL space and the Original space are available at https://app.jadbio.com/share/81be792e-3ba8-43e0-ace0-b8a4fe9657f0 and https://app.jadbio.com/share/32892dc1-739d-4b37-b6b5-50802587fb1b respectively.

**Fig. 11:**
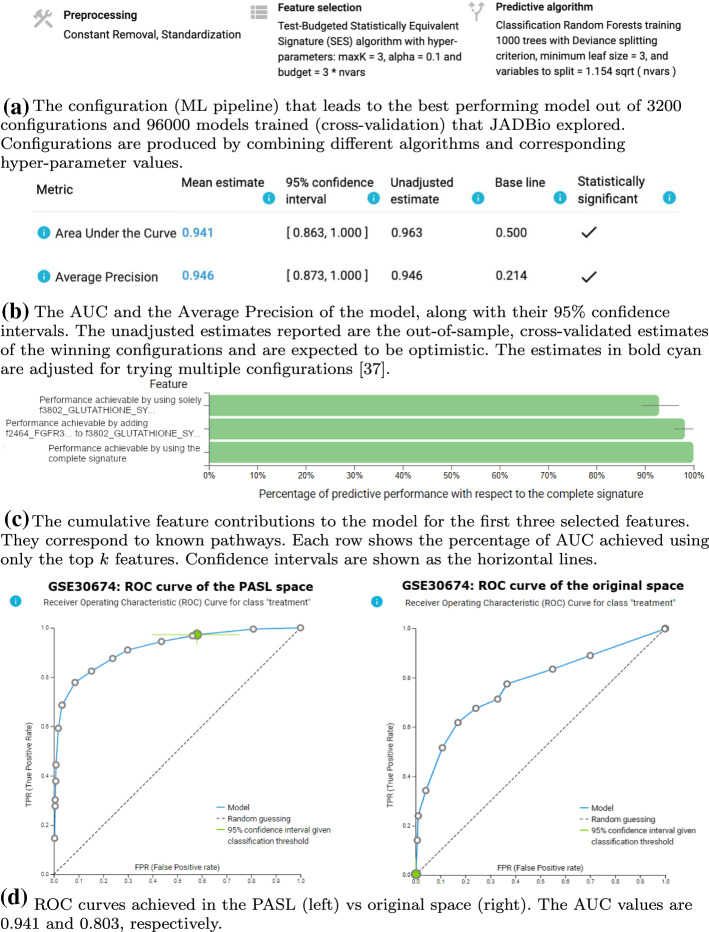
Predictive modeling with JADBio of of kinase inhibitor treatment vs treatment control of stimuli activated Jurkat T cells (dataset GSE30674). Panels a, b and c (left) were produced in the PASL space (https://app.jadbio.com/share/9463e28b-736c-4476-8d5f-b92d7364c6aa), panel c (right) in the original space (https://app.jadbio.com/share/fe02e291-7d35-4286-bdbe-2e600b1412a3)

### Identification of T cells subjected to different stimuli

Dataset GSE30674 contains the transcriptomics profile of 56 samples from an immortalized cell line of human T lymphocyte cells, namely Jurkat T cells. Samples were subjected to various stimuli and pathway inhibitors, with the aim of studying their effect on differentiation into Th1, Th2, Th17 or Treg phenotypes. The original study (Smeets et al., 2012) focused on studying how the differentiation of naive T cells is affected by the combination of stimuli and kinase inhibitors. The study did not attempt reporting molecular signatures. Particularly, their results underlined that three out of five kinase inhibitors had large effects on gene regulation and T cell differentiation, focusing particularly on the CCL1 and IL-2 mRNA induction. These results show that kinase activation is crucial for T cell differentiation. Consequently, for the present analysis we aim at identifying transcriptomics features that differentiate between the 12 samples (treatment control) subjected to differentiation stimuli (CD3, CD28, and PMA in various combination) in the presence of dimethyl sulfoxide (DMSO) and the 44 samples treated with combinations of the same differentiation stimuli in the presence of kinase inhibitors (treatment).

The analysis in the original feature space uses all the 54675 available mRNA values and achieves an AUC of 0.803 (0.589, 1.000) with Random Forest as best model. The analysis in the PASL space led to a higher predictive performance, AUC 0.941 (0.863, 1.000), with a more parsimonious Random Forest best model that uses only 3 features (outcomes are shown in Fig. 11a–c). Furthermore, 153 equivalent signatures were identified. This means that different features (pathway activation scores) could substitute for others in the selected features and still obtain an equally predictive model giving rising to different signatures. PASL’s selected features include “Reactome Gluthanione synthesis and recycling” in all signatures and “Reactome FGFR3B ligand biding and activation” in at least 5 of the equivalent signatures. Both pathways are associated with T cell activation. More specifically, gluthanione synthesis and recycling pathway is already correlated with T cell differentiation in mice (Lian et al., 2018). Furthermore, FGFR receptors’ family are associated with T Cell receptor signaling pathway (Byrd et al., 2003). These two pathways are not reported in the original publication but it is known that both of them interact with the MAPK, Lck/Cn and $PKC\theta$ pathways, which are found affected in the original study. These findings can be of interest regarding the selectivity of kinase inhibitors for future controlled differentiation of Jurkat T cells or even human blood. Figure 11d shows the ROC curves of the PASL space (left panel) and the Original space (right panel). The JADBio analyses both for the PASL space and the Original space are available in https://app.jadbio.com/share/9463e28b-736c-4476-8d5f-b92d7364c6aa and https://app.jadbio.com/share/fe02e291-7d35-4286-bdbe-2e600b1412a3 respectively.

## Discussion and conclusions

Molecular -omics and multi-omics data are notoriously high-dimensional. Their high-dimensionality impedes computational analysis as well as biological interpretation. To enable biological interpretation, biologists typically examine results at the level of known pathways, or genesets in general, not at the level of single gene expressions or other biomarkers. Several dimensionality reduction methodologies have been applied, or even have been specifically developed for -omics datasets. They have shown that -omics datasets can be indeed successfully compressed. However, general-type dimensionality reduction techniques end up with unintepretable feature spaces. The constructed features do not directly correspond to the well-organized biological knowledge in terms pathways and gene ontologies; they do not match the way biologists are used to examine results.

The Pathway Activity Score Learning or PASL algorithm is a dimensionality reduction technique that constructs features that directly correspond to known pathways (genesets) and they can be interpreted as pathway activity scores. It also constructs features that point to unknown pathways (genesets). PASL is relatively efficient and employs a greedy heuristic to create the next atom in its dictionary. In the paper, we compare PASL against a related technique, called PLIER, and show that PASL maintains more predictive information in the data. We also compare differential activation analysis to identify pathways that behave differently between two conditions against the standard gene set enrichment analysis. We show that PASL identifies affected pathways with higher statistical power (smaller *p* values) and is synergistic to GSEA.

Using a large collection of 50000 uniformly preprocessed samples spanning dozens of different diseases and PASL, we create a universal dictionary for the Affymetrix platform GPL570 to map any new dataset to constructed features of lower dimensionality (6000 vs 54675 features). We evaluate the power of the dictionary to maintain the predictive information across different tissues, organs, pathologies, and outcomes such as disease status, disease subtype, and response to treatment. The evaluation is performed on 165 test datasets of a total of 16286 samples. We use the AutoML platform JADBio that tunes the analysis pipelines in terms of algorithms and their hyper-parameters to deliver the best predicting model and conservative estimates of its out-of-sample performance. The models produced in PASL space are on average on par with the ones created on the original mRNA expressions space. In some cases however, they offer higher predictive performance and straightforward biological interpretation. The results suggest that analysis in PASL space could complement analysis in the original space.

Lastly, we would like to state few limitations of this work. The results in the test datasets have not been biologically examined in depth. A comparison against GSEA using the universal dictionary is also left as future work. The behavior of the algorithm with respect to available sample and its learning curve is not studied. The PASL algorithm does not have data-driven termination criteria for the construction of atoms. It does not produce statistical guarantees (i.e., *p* values) on the hypothesis that a given dictionary atom is not a random projection. The algorithm naively performs PCAs from scratch without cashing intermediate computations between iterations. Several other optimizations on the time complexity are also possible. Finally, PASL requires prior biological knowledge for its inference phase limiting the application of PASL on other collections of datasets. However, it is applicable in other domains, as long as there is available prior knowledge that can be expressed as a membership matrix.

## Data Availability

Available upon request.

## Footnotes

1 http://dataome.mensxmachina.org/.

2 http://www.affymetrix.com/support/technical/byproduct.affx?product=hg-u133-plus.

3 JADBio is often able to discover and return multiple signatures leading to model of statistically indistinguishable performance (Lagani et al., 2016)

## Code Availability

The code is available at https://github.com/mensxmachina/PASL
